# Supplementary material for: Exploring the Immunomodulatory Potential of Human Milk: Aryl Hydrocarbon Receptor Activation and Its Impact on Neonatal Gut Health
Source: Nutrients. 2024 May 19;16(10):1531. doi: 10.3390/nu16101531 (PMC11124328; doi:10.3390/nu16101531)
Supplement: Supplementary file 1 [file nutrients-16-01531-s001.zip › nutrients-2972148-supplementary.pdf]

nutrients

MDPI

*Supplementary Materials*

# Exploring the Immunomodulatory Potential of Human Milk: Aryl Hydrocarbon Receptor Activation and Its Impact on Neonatal Gut Health

**Naomi V. Wieser [1,*], Mohammed Ghiboub [1,2,3], Caroline Verseijden [1,2], Johannes B. van Goudoever [4], Anne Schoonderwoerd [4], Tim G. J. de Meij [2,5], Hendrik J. Niemarkt [6,7], Mark Davids [8], Antoine Lefèvre [9], Patrick Emond [9,10], Joep P. M. Derikx [3], Wouter J. de Jonge [1,2,11] and Bruno Sovran [1,3,12,*]**

**Material sand Methods:**

**Data Preparation and biomarker Analysis**

Data illustration and statistical analysis were performed with MetaboAnalyst5.0 (https://www.metaboanalyst.ca). The data were filtered (using the interquartile range method) to identify and remove noisy and non-informative variables that are unlikely to be of use when modeling the data as described prevously. Receiver-operating characteristic (ROC) analysis was also performed in MetaboAnalyst 5.0 as described previously using calculated normalized concentrations of 20 tryptophan metabolites. The area under the ROC curve (AUC) was calculated with 95% confidence intervals. Ratios between 2 tryptophan metabolite concentrations may yield more information than the 2 corresponding metabolite concentrations alone. Thus, we commanded MetaboAnalyst 5.0 to compute and include all possible ratios of the top 20 tryptophan metabolite pairs (based on $P$ values) to be integrated in further biomarker analysis. Individual metabolites and metabolite ratios with AUC≥0.8 and $p$<0.05 were selected. The final figures were adapted in Inkscape 0.92.4.

**DNA isolation for microbiome of human milk and formula**

Milk DNA was extracted following the Maxwell® RSC PureFood GMO and Authentication Kit, Promega (Wisconsin, USA). Briefly, the milk was weighed and then 1000μL CTAB buffer, 40 μL Proteinase K and 20 μL RNase A were added. Tubes were then vortexed and incubated in a shaker at 600rpm at 65C for 30 min. Samples were centrifuged at 14000rpm. Then 50 μL elution buffer is added to the elution tube. Next, 300 μL of Lysis buffer and 300 μL of the samples are added together into the tube of the Maxwell cartridge. DNA concentration was measured by nanodrop.

**Bacterial profiling of human milk and formula**

The 16S rRNA gene amplicons were produced using a single-step PCR procedure targeting the V3–V4 region, and this was carried out at the Microbiota Center Amsterdam (MiCA) as described earlier [48]. Amplified sequence variants (ASVs) were inferred for each sample individually with a minimum abundance of 4 reads [49]. Unfiltered reads were mapped against the collective ASV set to establish the abundances. Taxonomy was assigned using the IDTaxa [50] and SILVA 16S ribosomal database V132 [51].

**TEER measurements using ECIS**

Trans-endothelial electrical resistance (TER) was measured using an electric cell impedance sensing (ECIS) apparatus (Applied Biophysics, Troy, NY). HFOs were split into single cells and seeded at 100’000 cells/mL onto gold-coated electrode arrays (Applied Biophysics, Troy, NY) and grown to confluence. Then, the HFOs were treated with the

indoles and FICZ, and challenged with TNFα for 72h. Impedance was measured for the duration of the experiment. The data was normalized from the initiation of treatment and plotted as normalized TER.

**Supplementary figure and legends:**

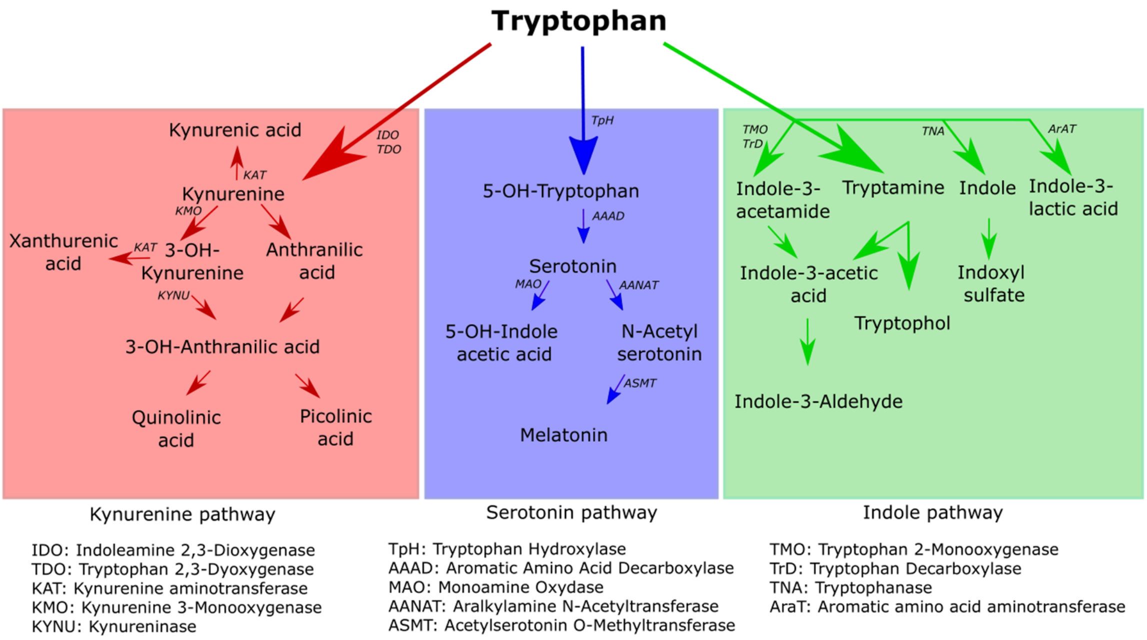


**Figure S1.** Overview of the Trp pathway. An overview and sequential metabolism of all measured metabolites across the three tryptophan (Trp) metabolic pathways.

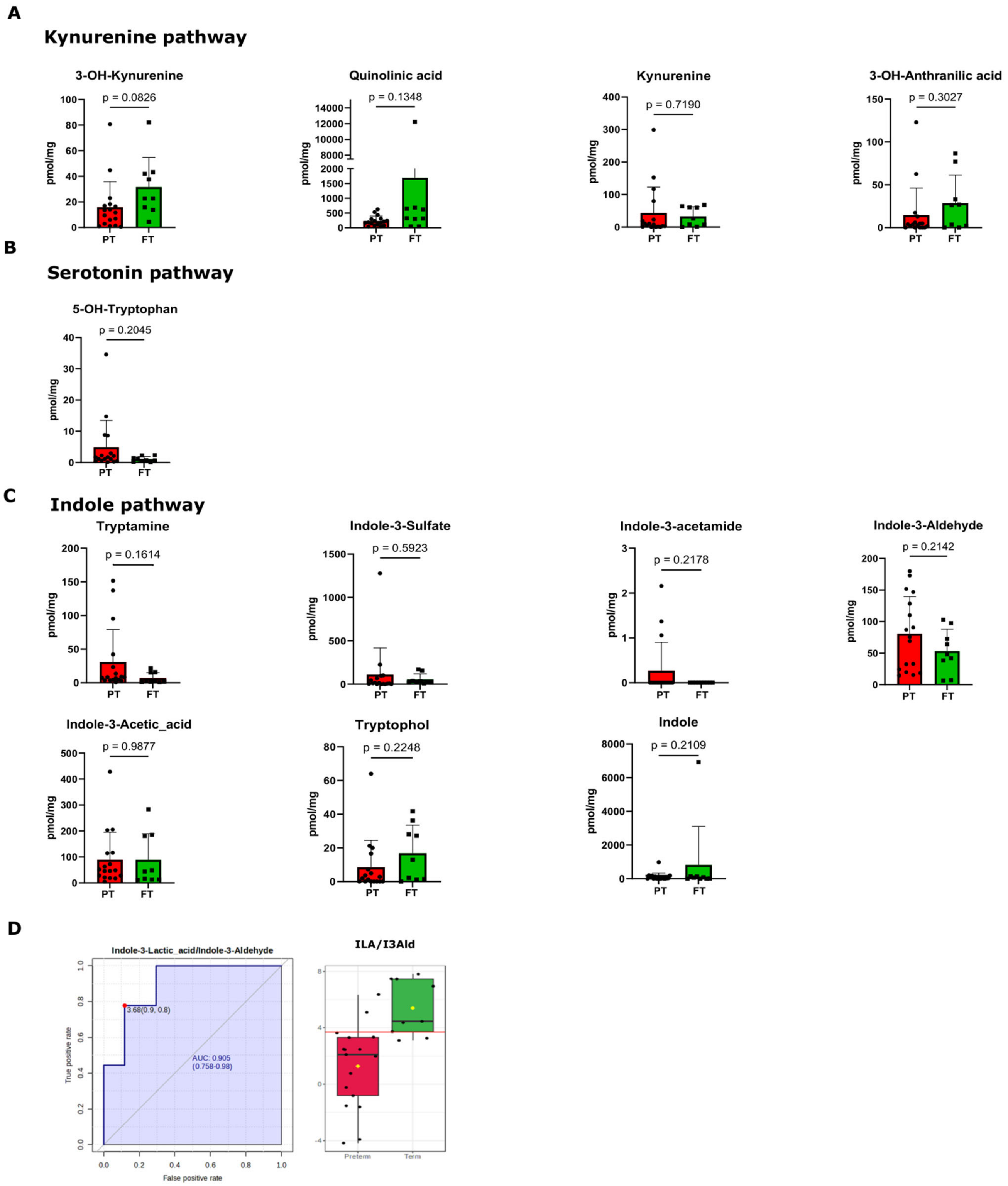


**Figure S2.** Remaining metabolites at birth in neonatal faecal samples. (**A**) Kynurenine metabolites which are not different between preterm and full-term at birth. (**B**) Serotonin metabolites which are no different between preterm and full-term neonates at birth. (**C**) Indole metabolites which are no different between preterm and full-term neonates at birth.

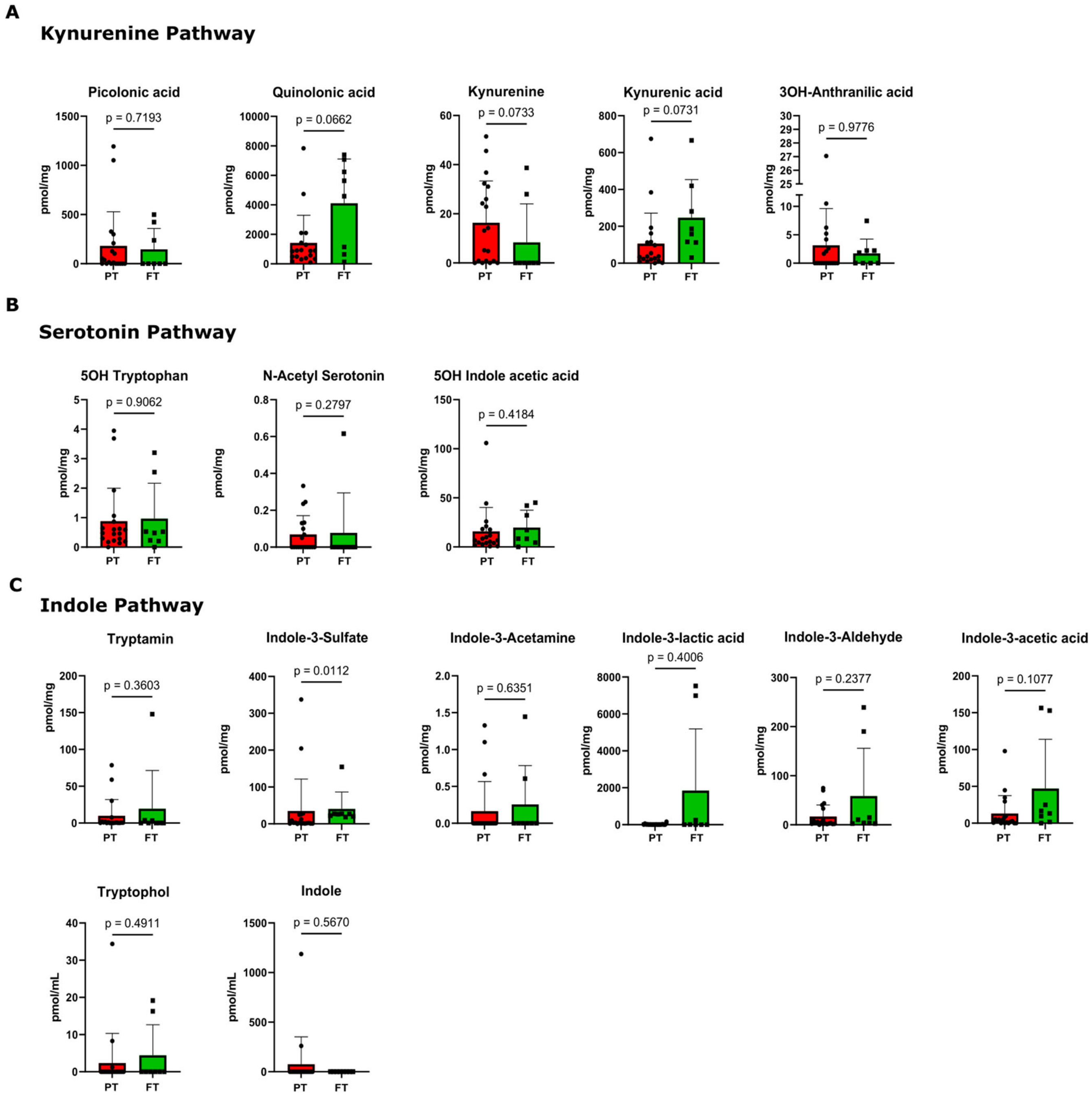


**Figure S3.** Remaining metabolites at 4 weeks in neonatal faecal samples. (**A**) Kynurenine metabolites which are no different between preterm and full-term neonates at birth. (**B**) Serotonin metabolites which are no different between preterm and full-term neonates at birth. (**C**) Indole metabolites which are no different between preterm and full-term neonates at birth. (**D**) Predictive metabolites to discriminate between preterm and full-term neonates. The receiver-operating characteristic (ROC) curves with their area under the ROC curve (AUC) scores and the associated box plot of raw values with their *P* value of the ILA-to-I3Ad.

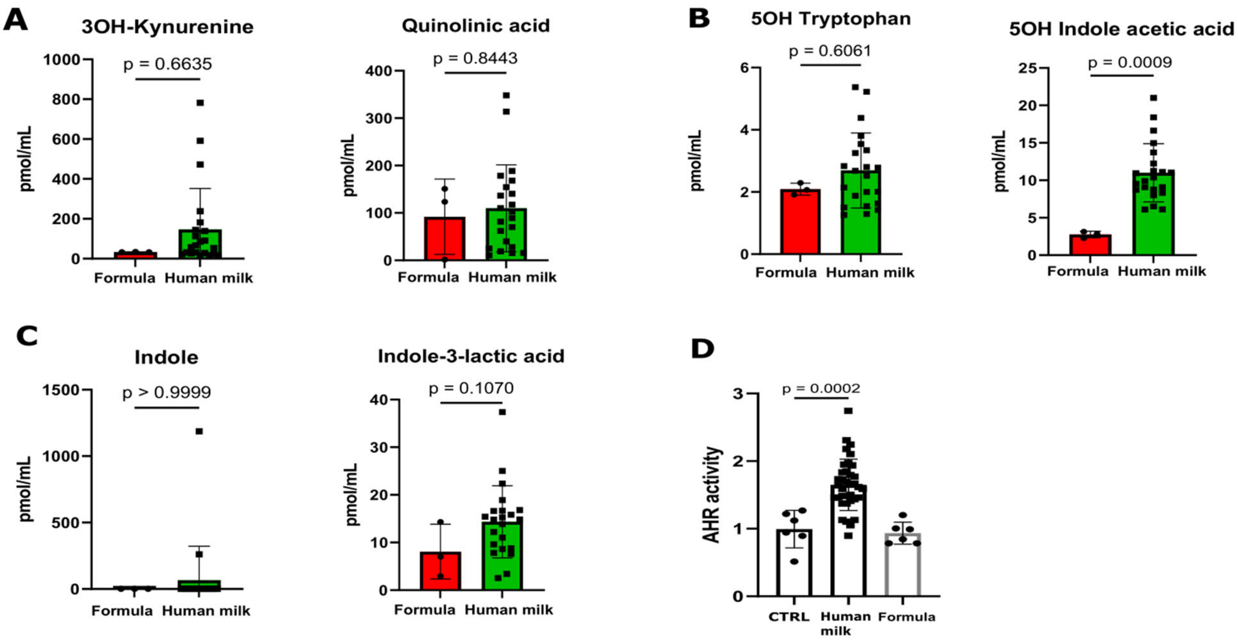


**Figure S4.** Remaining metabolites in human milk against formula milk. (**A**) Kynurenine metabolites which are no different between human and formula milk. (**B**) Serotonin metabolites which are no different between human and formula milk. (**C**) Indole metabolites which are no different between human and formula milk. (**D**) AHR activity of Human milk and formula.

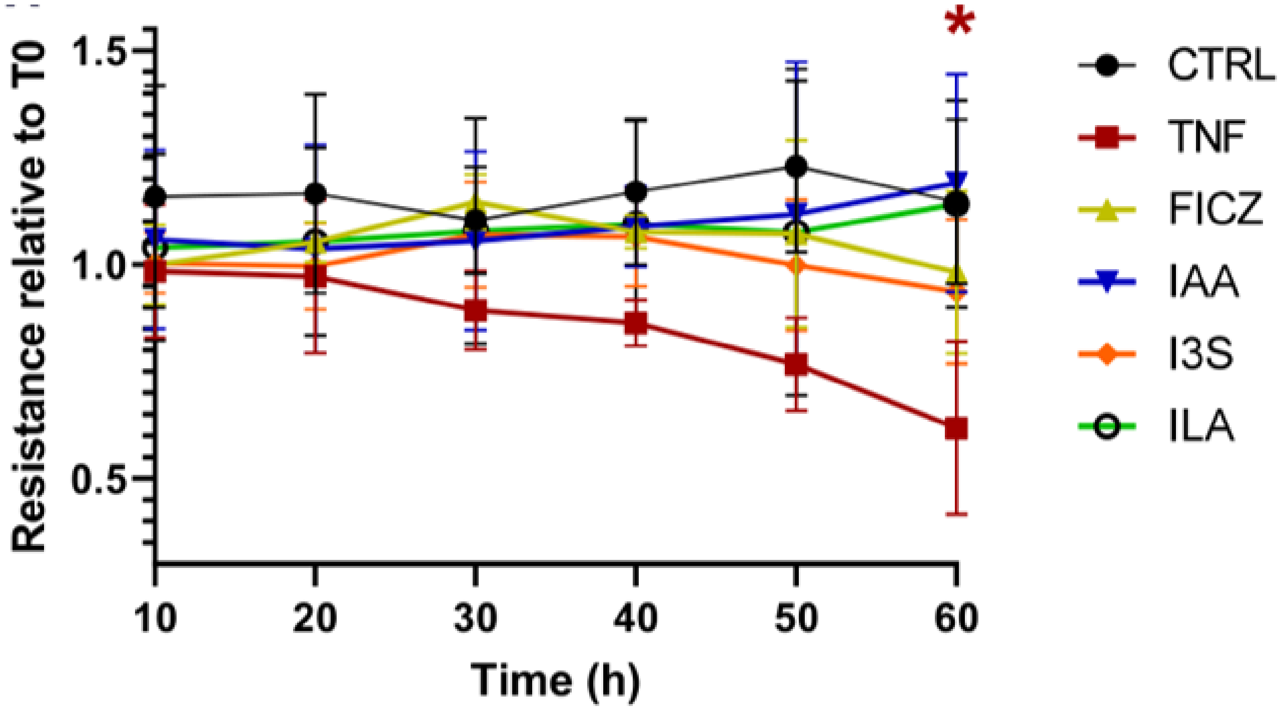


**Figure S5.** TEER measurements using the ECIS. TEER values relatively to T0 in 2D HFOs.
